# Glyphosate-induced changes in the expression of galanin and GALR1, GALR2 and GALR3 receptors in the porcine small intestine wall

**DOI:** 10.1038/s41598-024-59581-8

**Published:** 2024-04-17

**Authors:** Katarzyna Palus, Małgorzata Chmielewska-Krzesińska, Barbara Jana, Jarosław Całka

**Affiliations:** 1 Department of Clinical Physiology, Faculty of Veterinary Medicine, University of Warmia and Mazury, Oczapowskiego 13, 10-719 Olsztyn, Poland; 2 Department of Pathophysiology, Forensic Veterinary Medicine and Administration, Faculty of Veterinary Medicine, University of Warmia and Mazury in Olsztyn, Oczapowskiego 13, 10-719 Olsztyn, Poland; 3 Division of Reproductive Biology, Institute of Animal Reproduction and Food Research of the Polish Academy of Sciences, Tuwima 10, 10-078 Olsztyn, Poland

**Keywords:** Galanin, Galanin receptors, Glyphosate, Enteric nervous system, Small intestine, Neurophysiology, Enteric nervous system, Small intestine

## Abstract

Glyphosate is the active ingredient of glyphosate-based herbicides and the most commonly used pesticide in the world. The goal of the present study was to verify whether low doses of glyphosate (equivalent to the environmental exposure) evoke changes in galanin expression in intramural neurons in the small intestine in pigs and to quantitatively determine changes in the level of galanin receptor encoding mRNA (GALR1, GALR2, GALR3) in the small intestine wall. The experiment was conducted on 15 sexually immature gilts divided into three study groups: control (C)—animals receiving empty gelatin capsules; experimental 1 (G1)—animals receiving a low dose of glyphosate (0.05 mg/kg b.w./day); experimental 2 (G2)—animals receiving a higher dose of glyphosate (0.5 mg/kg b.w./day) orally in gelatine capsules for 28 days. Glyphosate ingestion led to an increase in the number of GAL-like immunoreactive intramural neurons in the porcine small intestine. The results of RT-PCR showed a significant increase in the expression of mRNA, which encodes the GAL-receptors in the ileum, a decreased expression in the duodenum and no significant changes in the jejunum. Additionally, intoxication with glyphosate increased the expression of SOD2-encoding mRNA in the duodenum and decreased it in the jejunum and ileum, but it did not affect SOD1 expression. The results suggest that it may be a consequence of the cytotoxic and/or neurotoxic properties of glyphosate and/or its ability to induce oxidative stress.

## Introduction

Along with the GAL-like peptide, alarin and GAL-message-associated peptide, galanin (GAL) is a neuropeptide of the GAL family^1^. GAL is an important neurotransmitter in the central and peripheral nervous systems, including the neurons that supply the gastrointestinal (GI) tract. The presence of GAL has been confirmed in the neurons of the dorsal motor vagal nucleus (DMX)^2^, the coeliac-superior mesenteric ganglion complex (CSMG)^3^, dorsal root ganglia (DRG)^4^ as well as in intramural ganglia—both myenteric and submucous in various sections of the GI tract) of many animal species and humans^5–7^. GAL plays a significant role in the control of the GI tract function; e.g. it regulates the intestine peristalsis, gastric juice secretion, and intestinal absorption and modulates inflammatory and neoplastic processes^1,5,6^. It performs its function owing to interactions with one of the three types of galanin receptors (GALRs): GALR1, GALR2 and GALR3, which are members of the G-protein-coupled receptor family^1^. The presence of GALRs has been demonstrated both in the central nervous system and in many peripheral tissues, including the GI tract^8,9^. It has been shown in many studies that GAL is an important neuroprotective factor, participating in the neural response to various pathological stimuli within the GI tract. GAL and GALRs expression has been shown to change in the course of gastric ulcer^10^, colitis^11,12^, injured axons in the intestine^12^, intoxication with acrylamide^7^, bisphenol A^13^ or systemic diseases, such as type I diabetes^14^.

The agricultural production intensification observed in recent years is linked to the widespread use of pesticides. These include glyphosate (N-(phosphonomethyl)-glycine)—the active ingredient of glyphosate-based herbicides (GBH) and, at the same time, the most commonly used pesticide in the world^15^. The widespread presence of glyphosate in groundwater, in precipitations and in soil results in its high levels in food products, such as beer, eggs, mineral water, oily seeds and vegetable oils, breakfast cereals, crackers and other cereal products and—in consequence—continuous exposure of humans and animals to this substance^16^. The herbicidal action of glyphosate is effected by the inhibition of 5-enolpyruvylshikimate-3-phosphate synthase and, in consequence, the biosynthesis of aromatic amino acids in plants^15^. Since the shikimic acid metabolic pathway does not occur in vertebrates, it was assumed that glyphosate would not have negative effects on animals or people^16^. However, many toxicology studies conducted during the past decade have confirmed the neurotoxic, teratogenic and carcinogenic effects of glyphosate^16–18^. Exposure to glyphosate leads to liver and kidney function disorders, endocrinal disorders and metabolic changes^17^. Studies using the rat as a research model showed that glyphosate administrated in low doses (approved for use in agriculture) induced endocrine effects and altered reproductive developmental parameters in both males and females^19^, impairs male offspring reproductive development by disrupting gonadotropin expression^20^ and alters gut microbial composition^21^. Glyphosate also has a negative impact on amphibians, causing a teratogenic effect on Xenopus laevis embryos incubated with 1/5000 dilutions of commercial glyphosate-based herbicides (GBH)^22^. Further, the neurotoxicity of glyphosate (administered in increasing doses) has been confirmed in studies using rodents^23^, while the genotoxicity was recorded in erythrocytes of broad-snouted caiman (*Caiman latirostris*) after in ovo exposure to high doses of glyphosate^24^. One of the main mechanisms responsible for glyphosate cytotoxicity is the ability to generate oxidative stress, i.e. excessive and unregulated production of reactive oxygen species^25^. The ability to induce oxidative stress has been confirmed both in aquatic animals and in mammals^25–28^. A low glyphosate concentration in water increased the level of reduced glutathione (GSH) in fish^25^ and rat livers^26^. Further studies of rats showed sublethal doses of glyphosate to increase malondialdehyde (MDA) levels in the liver^27^, whereas its long-term administration in low doses results in a higher level of MDA in the brain, plasma, kidneys and liver^28^. In effect, glyphosate intoxication leads to the activation of an antioxidative response expressed as an increase in the activity of antioxidative enzymes.

The International Agency for Research on Cancer classified glyphosate as being a probable human carcinogen (group 2A) in 2014^18^. There is an ongoing debate between scientists and regulatory bodies concerning a ban on glyphosate use in agriculture and safe doses of the substance for humans^16^. The European Food Safety Authority (EFSA) established that the acceptable daily intake (ADI) of glyphosate is 0.5 mg/kg b.w. per day^29^. Although glyphosate is poorly absorbed from the GI tract, it was shown that approx. 34% of glyphosate is in the small intestine of the rat, where a high level persists even seven days after ingestion^30^. Despite significant interest in glyphosate, its impact on the GI tract, including the enteric nervous system (ENS), has not been sufficiently identified. Having a large autonomy in controlling the GI tract function, the ENS is indisputably involved in the adaptation and defence processes in response to GI tract function disorders, referred to as neural plasticity^31^. Given the above, the objective of this study was to verify whether low doses of glyphosate (equivalent to the theoretical maximum daily intake (TMDI) and the acceptable daily intake (ADI) in Europe) bring about changes in galanin expression in intramural neurons in the small intestine in pigs and to quantitatively determine changes in the level of galanin receptor encoding mRNA (GALR1, GALR2, GALR3) in the small intestine wall.

## Results

**Figure 1:**
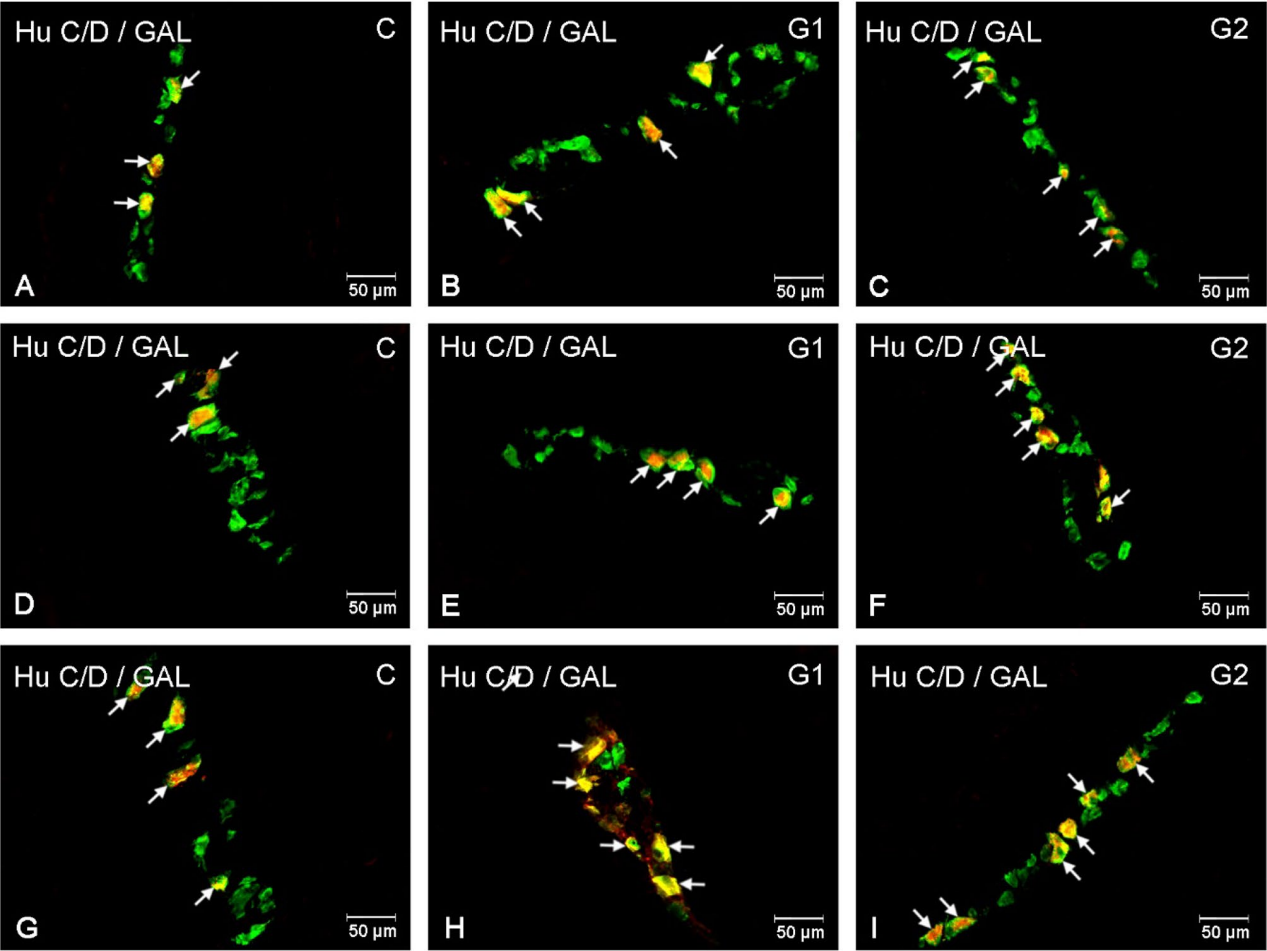
Distribution pattern of enteric nervous system neurons immunoreactive to HuC/D—used as a panneuronal marker and galanin (GAL) in the myenteric plexuses in the porcine small intestine in the control group (**A**, **D**, **G**), after lower (**B**, **E**, **H**) and higher doses (**C**, **F**, **I**) of glyphosate administration. Photographs (**A**–**C**) showing myenteric plexuses in the duodenum, (**D**–**F**) myenteric plexuses in the jejunum and (**G**–**I**) myenteric plexuses in the ileum. All photographs have been created by digital superimposition of two colour channels (green for HuC/D and red for GAL). Neurons immunoreactive to GAL are indicated with arrows.

**Figure 2:**
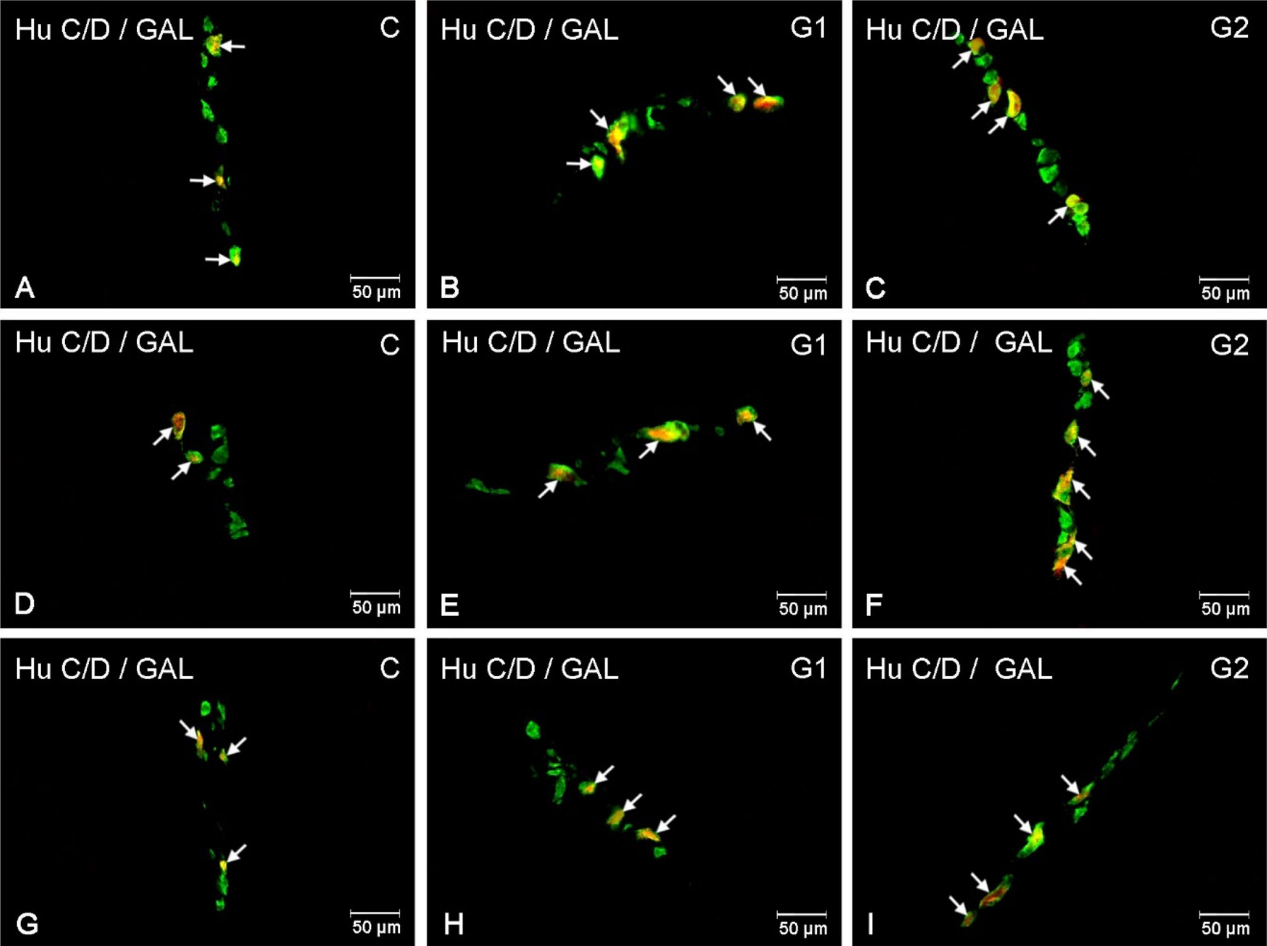
Distribution pattern of enteric nervous system neurons immunoreactive to HuC/D—used as a panneuronal marker and galanin (GAL) in the outer submucous plexuses in the porcine small intestine the control group (**A**, **D**, **G**), after lower (**B**, **E**, **H**) and higher doses (**C**, **F**, **I**) of glyphosate administration. Photographs (**A**–**C**) showing outer submucous plexuses in the duodenum, (**D**–**F**) outer submucous plexuses in the jejunum and (**G**–**I**) outer submucous plexuses in the ileum. All photographs have been created by digital superimposition of two colour channels (green for HuC/D and red for GAL). Neurons immunoreactive to GAL are indicated with arrows.

**Figure 3:**
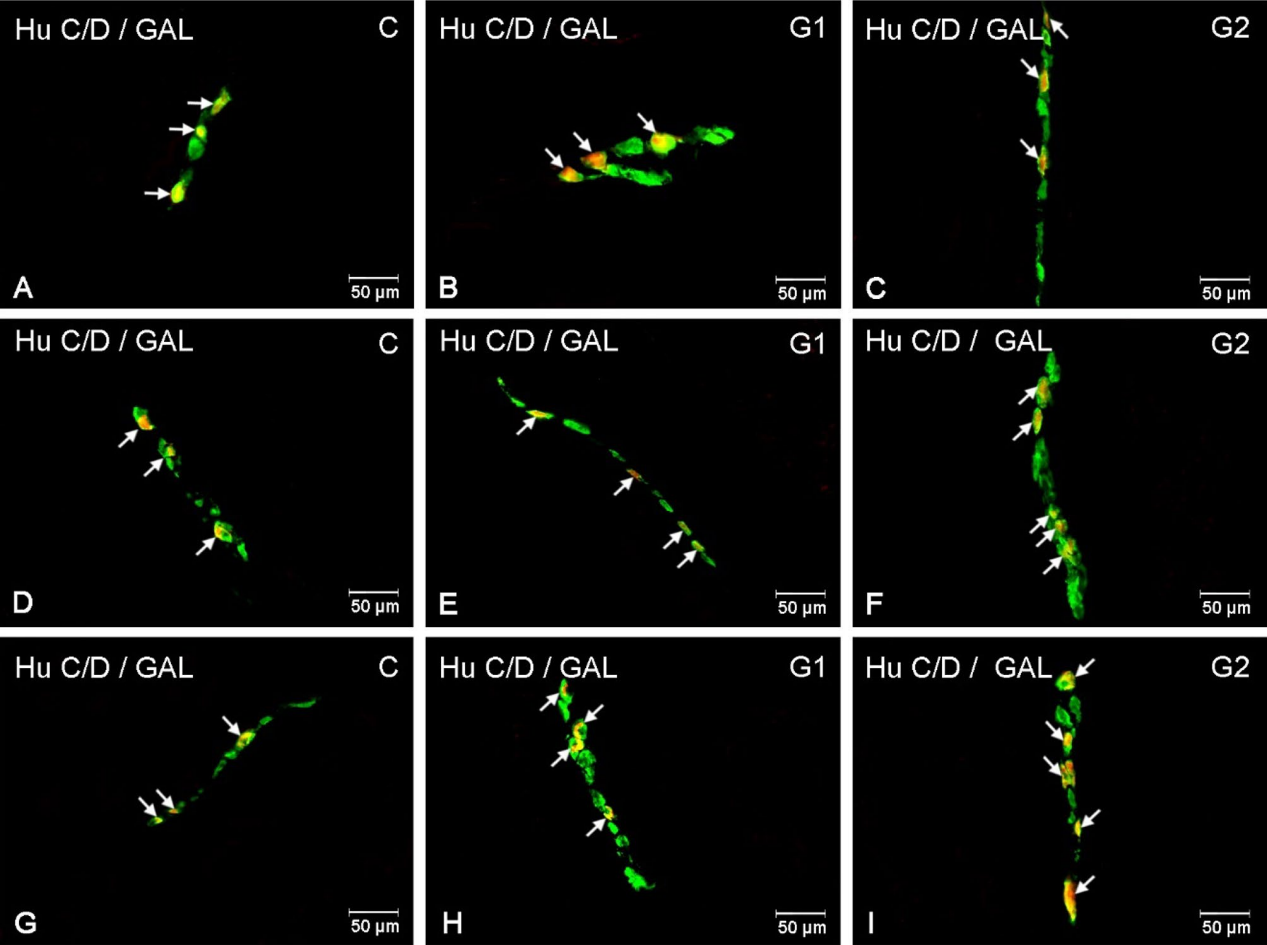
Distribution pattern of enteric nervous system neurons immunoreactive to HuC/D—used as a panneuronal marker and galanin (GAL) in the inner submucous plexuses in the porcine small intestine the control group (**A**, **D**, **G**), after lower (**B**, **E**, **H**) and higher doses (**C**, **F**, **I**) of glyphosate administration. Photographs (**A**–**C**) showing inner submucous plexuses in the duodenum, (**D**–**F**) inner submucous plexuses in the jejunum and (**G**–**I**) inner submucous plexuses in the ileum. All photographs have been created by digital superimposition of two colour channels (green for HuC/D and red for GAL). Neurons immunoreactive to GAL are indicated with arrows.

This study has shown that neurons in the ENS changed their neurochemical profile in response to glyphosate ingestion, showing an increase in galanin expression. Change intensity depended on the intestine section and the plexus type under examination (Table 1, Figs. 1, 2, 3). Regarding the myenteric plexus (MP), statistically significant changes in the number of GAL-like immunoreactive (LI) neurons in the duodenum and jejunum were found only in the group receiving the larger dose of glyphosate (increase from 3.65 ± 0.46% to 7.86 ± 0.39% and from 6.88 ± 0.41 to 16.51 ± 1.39%, respectively) (Fig. 1C,F). In the ileum, GAL-LI neurons accounted for 5.06 ± 0.56% of HuC/D-positive neurons in the control group (Fig. 1G) and glyphosate supplementation led to an increase in the number of GAL-positive neurons in both experimental groups: to 9.23 ± 0.40% in G1 (Fig. 1H) and to 15.88 ± 1.01% in G2 (Fig. 1I).

**Table 1:** The percentage of enteric nervous system (ENS) neurons immunoreactive to GAL in the porcine small intestine in control animals and after glyphosate intoxication.

	Myenteric plexus			Outer submucous plexus			Inner submucous plexus		
	C group	G1 group	G2 group	C group	G1 group	G2 group	C group	G1 group	G2 group
Duodenum	3.65 ± 0.46	4.35 ± 0.25	7.86 ± 0.39***	35.68 ± 1.35	38.00 ± 0.95	47.71 ± 0.87***	41.48 ± 1.59	47.87 ± 1.53*	64.81 ± 1.58***
Jejunum	6.88 ± 0.41	7.48 ± 0.44	16.51 ± 1.39***	36.56 ± 1.62	43.57 ± 2.13*	45.20 ± 1.06***	48.56 ± 1.52	52.35 ± 1.26	61.06 ± 1.04***
Ileum	5.06 ± 0.56	9.23 ± 0.40**	15.88 ± 1.01***	38.46 ± 1.36	43.33 ± 1.38*	53.54 ± 1.18***	48.14 ± 1.77	55.09 ± 0.80**	67.41 ± 1.09***

Three thresholds (*p < 0.05, **p < 0.01, and ***p < 0.001) were used to indicate statistically significant differences.

In the outer submucous plexus (OSP), only supplementation of the larger dose brought about significant changes in GAL expression in the duodenum: an increase in the number of GAL-positive neurons from 35.68 ± 1.35 to 47.71 ± 0.87% (Fig. 2A,C). In turn, significant differences in the jejunum were observed in the gilts receiving both glyphosate doses: increase from 36.56 ± 1.62% in control to 43.57 ± 2.13% in G1 and to 45.20 ± 1.06% in G2 (Fig. 2D–F). Similarly, in the ileum, an increase in the GAL-LI neuron population was observed in the gilts receiving both glyphosate doses: from 38.46 ± 1.36% in the control group to 43.33 ± 1.38% in G1 and 53.54 ± 1.18% in G2 (Fig. 2G–I).

A similar trend was observed in the inner submucous plexus. Both glyphosate doses brought about significant changes in the duodenum: from 41.48 ± 1.59% in the control to 47.87 ± 1.53% in G1 and to 64.81 ± 1.58% in G2 (Fig. 3A–C). The increase in the jejunum was significant only in G2: from 48.56 ± 1.52% to 61.06 ± 1.04% (Fig. 3D,F). In the ileum, an increase in GAL-immunoreactivity was significant in both experimental groups: from 48.14 ± 1.77% in control to 55.09 ± 0.80% in G1 and to 67.41 ± 1.09% in G2 (Fig. 3G–I).

**Figure 4:**
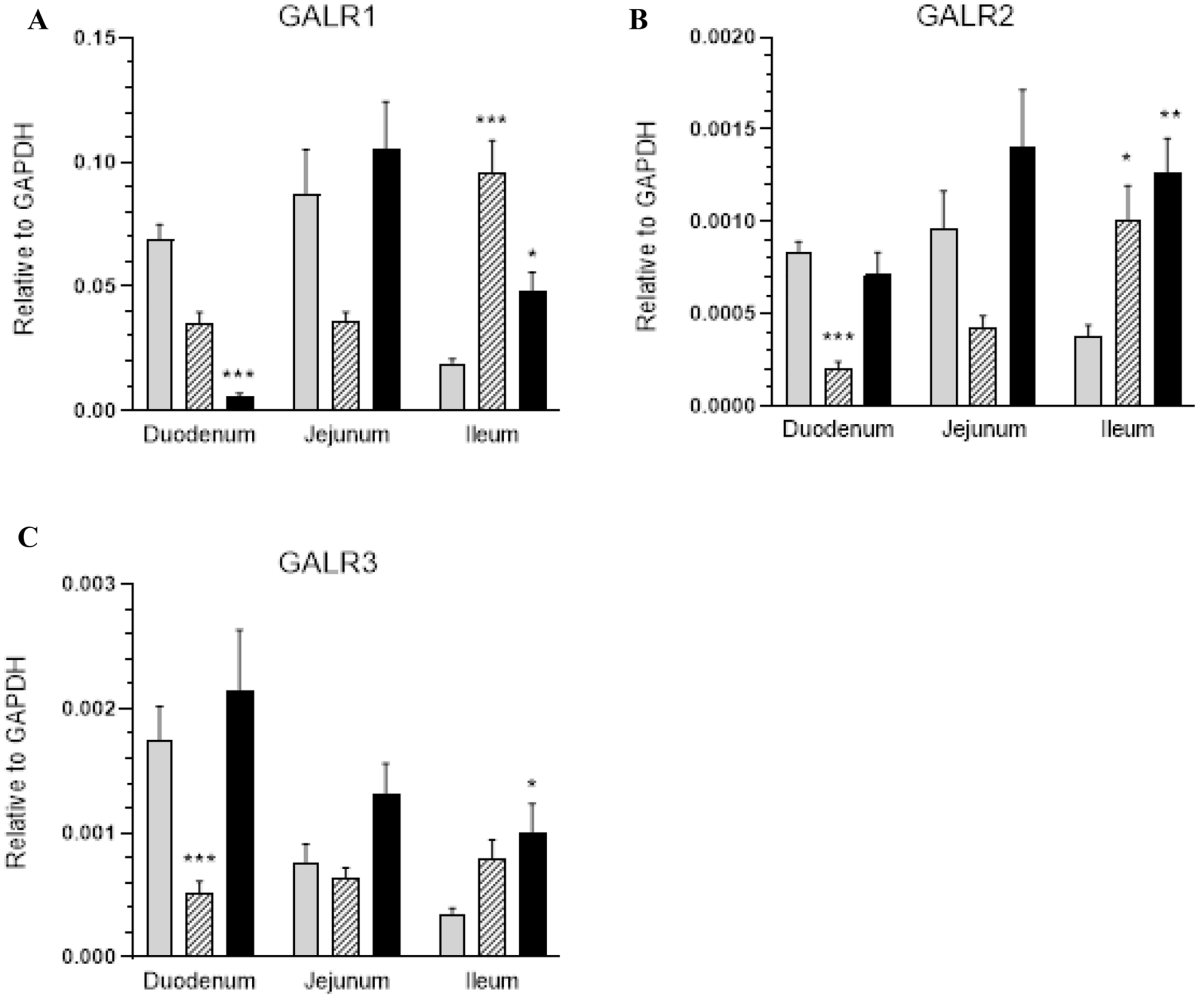
Expression of mRNA encoding GALR1, GALR2, GALR3 in the small intestines tissue. Expression of GALR1 (**A**), GALR2 (**B**) and GALR3 (**C**) mRNA in the duodenum, jejunum and ileum collected from the control (grey bars), G1 (hatched bars) and G2 (black bars) groups. Levels of GALR1, GALR2 and GALR3 mRNA were measured by Real-Time PCR. The data obtained from each sample were normalized to GAPDH. Relative quantities (RQ) of mRNA were analysed using the comparative Ct method. Each cDNA sample was amplified in triplicate and all data are expressed as the mean ± SEM, *p < 0.05, **p < 0.01, ***p < 0.001.

**Figure 5:**
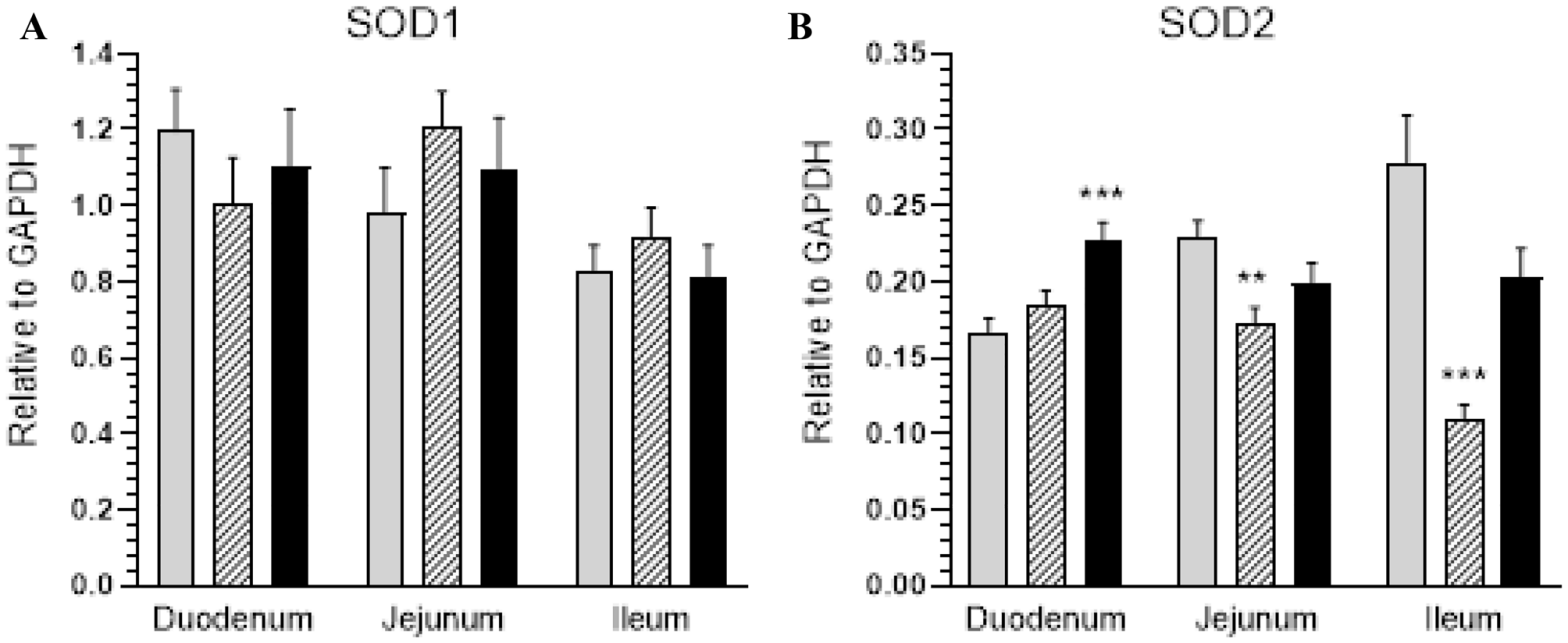
Expression of mRNA encoding SOD1 and SOD2 in the small intestines tissue. Expression of SOD1 (**A**) and SOD2 (**B**) mRNA in the duodenum, jejunum and ileum collected from the control (grey bars), G1 (hatched bars) and G2 (black bars) groups. Levels of SOD1 and SOD2 mRNA were measured by Real-Time PCR. The data obtained from each sample were normalized to GAPDH. Relative quantities (RQ) of mRNA were analysed using the comparative Ct method. Each cDNA sample was amplified in triplicate and all data are expressed as the mean ± SEM, **p < 0.01, ***p < 0.001.

The results of RT-PCR showed a significant increase in the expression of mRNA which encodes the GALR1 receptor (in G1 and G2) (Fig. 4A), GALR2 receptor (in G1 and G2) (Fig. 4B) and GALR3 receptor (in G2) (Fig. 4C) in the ileum. The mRNA receptor expression in the duodenum was found to decrease: GALR1 in G2 (Fig. 4A), GALR2 receptor in G1 (Fig. 4B) and GALR3 in G1 (Fig. 4C). The expression of galanin receptors in the jejunum did not change significantly (Fig. 4A–C). Intoxication with glyphosate also increased the expression of SOD2-encoding mRNA in animals receiving higher dose of glyphosate in the duodenum (Fig. 5B) and decreased it in the G1 group in both the jejunum and ileum (Fig. 5B). However, glyphosate in both doses did not affect SOD1 expression in all fragments of intestines investigated (Fig. 5A).

## Discussion

This study was the first to demonstrate that intoxication with low doses of glyphosate changed GAL expression in intramural neurons and galanin receptors in the small intestine wall of the pig. Due to comparable physiological processes, intestinal flora, and anatomy similar to humans, the pig is a useful animal model for studying intestinal pathology^32^, and thus, the obtained results can be referred to human medicine. Studies conducted to date have shown that GAL participates in the regulation of the GI tract function in mammals and in humans, not only in the physiological state but also in the regulation of pathological processes. Inflammatory processes in the intestines, such as proliferative enteropathy^33^, *Brachyspira hyodysenterieae* infection^11^, experimentally and naturally induced colitis^12^, or gastric ulcers^10^, had a considerable impact on the GAL level and increased its expression. Likewise, long-term NSAID (aspirin, naproxen and indometacin) supplementation increased GAL expression in all intestinal ganglia in pig duodenum and jejunum^34,35^. The same effect was observed as a result of pig intoxication with bisphenol A^13^ and acrylamide^7^. The increased density of GAL-containing fibres was also observed in the duodenum of people with alcoholic disease^36^. Further, diabetes, which often involves intestinal peristalsis disorders, has led to an increase in the number of intestinal neurons in the pig small intestine^37^, whereas it decreased the GAL concentration in the gastrointestinal tract in mice^38^. An increase in the population of GAL-positive neurons following glyphosate intoxication, as observed in this experiment, and earlier studies of the role of GAL in pathological processes in the GI tract, suggest that GAL may be an important factor involved in adaptive or neuroprotective processes of intestinal neurons, known for their neuroplastic properties expressed as a change of their neurochemical nature.

Earlier studies have shown that GALRs mRNA expression is high in the GI tract of many animal species and in humans. The presence of the GALR1 receptor has been reported in the muscularis externa, mucosal membrane, on ENS neurons in the GI tract of the mouse^39^, rat^40^, guinea pig^41^, pig^10,11^ and humans^42^. GAL decreases the peak pressure and contractions of longitudinal muscles, inhibiting intestinal peristalsis by GALR1 stimulation^43^. Moreover, GALR1 activation brings about the inhibition of voltage-dependent Ca^2+^ influx in a culture of rat muscularis externa neurons^44^. Further, the participation of the GALR1 receptor in motility and secretion control in the GI tract is largely associated with the regulatory impact on the secretion of other neurotransmitters^45^. Changes in GALR1 expression were observed in CNS neurons and in many peripheral tissues, including the GI tract, in many inflammatory and neoplastic processes^10,39,42^. Kiezun et al.^42^ suggest that GALR1 participates in the modulation of cancer cell proliferation in people with colon cancer. Increased GALR1 expression was observed in inflammatory processes in the GI tract, including experimentally induced antral ulcers in pigs^10^, dextran sulphate sodium-induced colitis in mice^39^ and Crohn’s disease in humans^46^. This confirms that GALR1 is a key element of communication between the immune and neuroendocrine systems of the intestine. In turn, the findings of the study conducted by Wąsowicz et al.^11^ were different. Namely, decreased GALR1 expression was observed in *Brachyspira hyodysenteriae*-induced colitis in pigs. The authors explain this discrepancy by extensive mechanical damage of tissues caused by inflammation or a different role of GAL in this part of the GI tract. The second hypothesis can also explain the findings of this experiment. Glyphosate supplementation decreased GALR1 expression in the group receiving higher doses in the duodenum and increased expression in both experimental groups in the ileum. This may result from the different functions of these intestine sections. The duodenum is the part of the GI tract where the food digestion process is the most intense and, at the same time, the concentration of glyphosate contained in the food is the highest. Whereas, ileum, with its concentration of lymphatic tissue, participates in the local inflammatory response and has thinner wall, with more narrow walls and fewer blood vessels. Food spends most of the time in it so it have the longest contact with glyphosate contained in food^47^.

GALR2 mRNA expression was also confirmed in all the GI tract sections, mainly in the muscularis externa, on myenteric neurons and sensory nerves^8–11,48,49^. The receptor’s location is linked to its function—regulation of the GI tract peristalsis, modulation of the sensory signal and response to pain^48,49^. Changes in the receptor expression in the GI tract occurred in inflammation and axotomy^49^. Given the increase in the receptor expression in the ileum wall and an increase in the GAL-LI neuron population in the myenteric plexus in both groups, it is suspected that peristalsis of this part of the small intestine increases in response to glyphosate supplementation. However, further research is necessary to confirm this hypothesis.

Although GALR3 distribution has been confirmed both in muscularis externa and on myenteric neurons, mRNA expression is considerably lower than the other galanin receptors^8–11,50^. Not much is also known about the function of this receptor in the GI tract. There have only been reports on GALR3 participation in the control of oedema formation and microcirculation regulation in dermatitis^51^. It may play a similar role in the regulation of local blood circulation and the inflammation process in the intestine, but this requires further research.

The mechanism of glyphosate’s toxic effect on mammalian tissues, including ENS, has not been elucidated. Studies of the harmful effects of glyphosate-based herbicides on the GI tract have shown it to cause histopathological changes, change the expression profile of genes involved in biotransformation and the level of pro-inflammatory cytokines in the chicken liver and small intestine^52^, damage the muscularis externa in gastric glands^53^. It also leads to hepatocyte degeneration in rats^53^, damages epithelial cells by the loss of the paracellular barrier in tight connections in the human intestinal epithelial cell line^54^, and causes injuries in the upper GI tract in humans^55^. Previous research demonstrated that oxidative stress is one of the main mechanisms responsible for glyphosate cytotoxicity^25–28^. In the present study, the antioxidant potential was evaluated through the expression of SOD mRNA in the intestinal wall. An increase in the expression of SOD2-encoding mRNA in the duodenum is consistent with earlier studies of the species, where an increase in SOD and catalase (CAT) activity was observed in the duodenum of piglets receiving glyphosate with fodder^56^. On the other hand, a decrease in SOD2 expression in the jejunum and ileum is consistent with the findings of a study on rats^28^. This suggests that glyphosate disrupts the antioxidant protection system in the intestines. However, further studies focusing on the activity of antioxidant enzymes and the production of free radicals are needed to confirm that oxidative stress is responsible for the changes observed in the present study. Moreover, oxidative stress is also the main mechanism of glyphosate neurotoxicity in the CNS and an important risk factor for neurodegenerative diseases^23^. Its harmful effect on the nervous system is well documented, especially for the CNS, where it inhibits enzyme activity, blocks cell proliferation, and disrupts the serotoninergic, noradrenergic and dopaminergic system function, leading to changes in neurotransmitter levels^23,57,58^. It has also been shown to lead to a change in the neurochemical profile of ENS neurons^59^. Taking into account the well-known neuroprotective function of GAL in the ENS^5,12^, we may speculate that a GAL expression increase and changes in its receptor expression, as observed in the current study, are associated with the neuroprotective function of the peptide, activated in response to glyphosate supplementation.

It cannot be ruled out that changes observed in the present study may be the result of intestinal microflora disorders caused by glyphosate. According to the findings of earlier studies, glyphosate considerably changes the composition of intestinal bacteria, leading to dysbiosis and neurobehavioral changes^21,60^. The impact of changes in intestinal microflora on the ENS has been documented earlier^61^.

It should be emphasized that further research is necessary to elucidate the mechanisms of the toxic effect of glyphosate on the GI tract and ENS neurons. Measuring the level of markers of neuronal damage may contribute to confirmation of the neurotoxic effect of glyphosate on ENS neurons. However, the determination of the level of antioxidant enzymes (superoxide dismutase, catalase, glutathione peroxidase) or the level of lipid peroxidation, protein and/or DNA oxidation can clearly answer the question of whether the cytotoxic effect of glyphosate in the intestines is due to its induction of oxidative stress.

In summary, this study was the first to show that low doses (acceptable for intake) of glyphosate have an impact on the number of intestinal galaninergic neurons and the expression of galanin receptors in the pig small intestine wall. Although the mechanism of the toxic effect of glyphosate on the ENS has not been elucidated, the data supported by earlier reports suggest that it is a consequence of the cytotoxic and/or neurotoxic properties of glyphosate or its ability to induce oxidative stress. However, further research is needed to elucidate all aspects of glyphosate's impact on the ENS. Given that only low glyphosate doses have been applied in this study, there is a topical question of whether current legal regulations are sufficient to protect consumer health.

## Materials and methods

### Experimental procedures and tissue collection

All of the procedures on animals were conducted in compliance with the relevant Polish and EU regulations in the field of Animal Protection and Welfare (Leg. Decree 26/2014 implementing EU directive 2010/63/EU) and were approved by the Local Committee for Animal Experiments in Olsztyn (Approval No. 62/2020). The experiment was conducted on 15 sexually immature gilts (8 weeks, approx. 20 kg b.w.) divided into 3 study groups (5 animals in each): ((1) control (C)—animals receiving empty gelatin capsules, (2) experimental 1 (G1)—animals receiving a low dose of glyphosate (analytical standard, purity > 99.5%, Sigma-Aldrich, CAS-No.: 1071-83-6)—equivalent to the theoretical maximum daily intake (TMDI) in Europe—0.05 mg/kg b.w./day^29^, (3) experimental 2 (G2)—animals receiving a higher dose of glyphosate—equivalent to the acceptable daily intake (ADI)—0.5 mg/kg b.w./day^29^ orally in gelatine capsules for 28 days during the morning meal. The animals were weighed once a week during the experiment to establish the right glyphosate dose. After supplementation, all gilts were treated with azaperone (Stresnil, Jansen Pharmaceutica N.V., 4 mg/ kg of body weight, i.m.) and after 15 min euthanized using a lethal dose of sodium pentobarbital (Morbital, Biowet Puławy; 0.6 ml/kg of body weight, i.v.). Subsequently, sections of the small intestine (duodenum, jejunum, ileum) were collected for further examination. The intestine sections intended for immunofluorescence staining were fixed in a 4% buffered paraformaldehyde solution (pH 7.4) for one hour. The tissues were washed for three days in phosphate-buffered saline (PBS, pH 7.4) and ultimately immersed in 30% sucrose solution and kept at the temp. of 4 °C. The collected intestine sections (duodenum, jejunum, ileum) for Real-Time PCR were fixed in RNAlater® (Ambion, Austin, TX, USA) and kept overnight at − 4 °C, and subsequently frozen to − 80 °C and kept until the analysis.

### Double immunofluorescent staining

The 12 µm-thick frozen duodenum, jejunum and ileum sections were doubly stained (as described previously by Palus et al.^52^ with a mixture of primary antibodies against nerve cell marker HuC/D (mouse polyclonal, Invitrogen, Waltham, MA, USA, Cat. No. A-21271, working dilution: 1:1000) and galanin (GAL, rabbit, cat. No. RIN7153, Peninsula, San Carlos, CA, USA, working dilution 1:3000) and the corresponding secondary antibodies (Alexa Fluor 488 nm donkey anti-mouse, ThermoFisher Scientific, Waltham, MA, USA; Cat. No. A21202; working dilatation: 1:1000 and Alexa Fluor 546 nm goat anti-rabbit, ThermoFisher Scientific, Waltham, MA, USA, Cat. No. A11010, working dilution: 1:1000). The specificity control for the antibodies used in the tests included a test of omission, replacement and pre-adsorption. Sections distanced by at least 100 µm were taken for staining to avoid analysing the same neurons. Neurons immunoreactive towards GAL were counted and photographed under an epifluorescence microscope (Olympus BX51). The population of GAL-positive neurons was counted in each of the enteric plexuses (myenteric plexus (MP), outer submucous plexus (OSP), inner submucous plexus (ISP)) in each small intestine section as a percent of Hu C/D-positive neurons. At least 500 HuC/D-positive neurons were counted for each plexus type.

### Real-time PCR

Total RNA was isolated from 50 µg of tissue homogenate (perpendicular section covering all layers (serosa, muscularis propria, submucosa and mucous membranes) of the intestine) (GeneJET™ RNA, cleaning kit, Thermo Fisher Scientific, Waltham, MA) as per the manufacturer’s protocol. Subsequently, the samples were evaluated qualitatively and quantitatively (Spectrophotometer NanoVue®, Thermo Fisher Scientific, Waltham, MA) and reverse transcription of mRNA to cDNA was performed (Maxima First Strand cDNA Synthesis Kit for RT-qPCR, Thermo Fisher Scientific, Waltham, MA). The reverse transcription was performed in a Biometra Thermocycler (Biometra T Gradient Professional Basic) for 10 min at 25 °C, 30 min at 65 °C, 5 min at 85 °C, and subsequently kept at 4 °C for 10 min. All samples were frozen at − 80 °C.

Six genes were examined in each cDNA sample: GALR1, GALR2, GALR3, SOD 1, SOD 2 and porcine glyceraldehyde 3-phosphate dehydrogenase (GAPDH) as the housekeeping gene in three replicates on a 96-well plate. The primers were designed with Primer-BLAST (http://ncbi.nlm.nih.gov), and their precise description can be found in an earlier publication^11^. Table 2 shows the primers used in the study. The PCR was performed in the 7500 fast real-time PCR system (Applied Biosystems, Waltham, MA, USA) with a thermal profile comprising: pre-denaturation 10 min at 95 °C, denaturation 15 s at 95 °C and hybridisation 1 min at 60 °C for 40 cycles.

**Table 2:** Sequences of primers used in real-time PCR.

Gene	Sequences of primers	Sequence of origin (in gene bank)
GAPDH	Forward: TTCCACCCACGGCAAGTT	NM_001206359.1
GAPDH	Reverse: GGCCTTTCCATTGATGACAAG	NM_001206359.1
GALR1	Forward: AGGATCACGGCGCACTGCCT	XM_003480426.2
GALR1	Reverse: GGGATTCCTTGCCAATGTGGCACT	XM_003480426.2
GALR2	Forward: GCCAAGCGCAAGGTAACGCG	XM_003484313.1
GALR2	Reverse: GTAGGTGGCGCGGGTAAGCG	XM_003484313.1
GALR3	Forward: GCACCACGCGCTCATCCTCT	XM_003355348.2
GALR3	Reverse: AGACCAGCGGGTTGAGGCAG	XM_003355348.2
SOD1	Forward: AGGATCAAGAGAGGCACGTTG	NM_001190422.1
SOD1	Reverse: TTGTGCGGCCAATGATGGA	NM_001190422.1
SOD2	Forward: CGGTGGAGGCCACATCAAT	NM_214127.2
SOD2	Reverse: GCTTCCAGCAATTCCCCTTTG	NM_214127.2

The primers were designed using sequences of origin available in Gen Bank and Primer-BLAST software (http://ncbi.nlm.nih.gov).

### Statistical analysis

The data from immunofluorescence staining and RT-PCR analysis were determined to be normally distributed using the D’Agostino and Person omnibus normality test using Statistica 13 software. Determinations of differences were made using a one-way analysis of variance (ANOVA) with the Dunnett test and presented as a mean ± SEM.

### Ethical approval

The studies presented in the manuscript were carried out in accordance with the ARRIVE guidelines. All study procedures were approved by the Local Ethics Committee for Experiments on Animals (University of Warmia and Mazury in Olsztyn, Poland; Approval No. 62/2020). The guidelines in EU Directive 2010/63/EU for animal experiments were included.

## Data Availability

The datasets used and/or analysed during the current study available from the corresponding author on reasonable request.
